# Polyphenolic Profile and Bioactivity Study of *Oenothera speciosa* Nutt. Aerial Parts

**DOI:** 10.3390/molecules14041456

**Published:** 2009-04-07

**Authors:** Mohamed S. Marzouk, Fatma A. Moharram, Rabab A. El Dib, Siham M. El-Shenawy, Ahmed F. Tawfike

**Affiliations:** 1 Natural Products Group, Centre of Scientific Excellence, National Research Center, El-Behoos St. 31, Dokki, Cairo, Egypt; 2 Department of Pharmacognosy, Faculty of Pharmacy, Helwan University, Cairo, Egypt; E-mails: famoharram1@hotmail.com (F-A.M.); reldib@yahoo.com (R-A.ED.); ahmedfares82@hotmail.com (A-F.T.); 3 Department of Pharmacology, National Research Center, El-Behoos St.31, Dokki, Cairo, Egypt; E-mail: siham_elshenawy@yahoo.com (S-M.ES.)

**Keywords:** *Oenothera speciosa*, Biflavonol, Antihyperglycaemic, Anti-inflammatory, Antioxidant.

## Abstract

Two new flavonol glycosides, myricetin 4'-*O*-α-l-rhamnopyranoside (**1**) and quercetin 3'-*O*-α-l-rhamnopyranoside (**2**), together with a novel biflavonol compound, speciin (**3**), as well as eleven phenolic metabolites, namely myricitrin (**4**), europetin 3-*O*-α-l-^1^C_4_-rhamnopyranoside (**5**), quercitrin (**6**), hyperin (**7**), rhamnetin 3-*O*-β-galacto-pyranoside (**8**), caffeic acid (**9**), caffeic acid methyl ester (**10**), chlorogenic acid (**11**), chlorogenic acid methyl ester (**12**), gallic acid (**13**) and gallic acid methyl ester (**14**), were identified from the 80 % methanol extract of the aerial parts (leaves and stems) of *Oenothera speciosa* Nutt. (Onagraceae). In addition myricetin (**15**), quercetin (**16**) and ellagic acid (**17**) were identified from the chloroform extract. The structures were established depending on their chemical and physical analyses (UV, HR-ESIMS, 1D and 2D NMR). It was found that 80 % aqueous methanol extract of *O. speciosa* is non-toxic to mice up to 5 g kg^-1^b.wt. The investigated extract exhibited significant antihyperglycaemic and anti-inflammatory activities in a dose dependant manner. Also, the 80 % methanol extract, myricitrin (**4**) and hyperin (**7**) showed potent antioxidant activity *in vitro* using 1,1-diphenyl 2-picryl hydrazyl (DPPH) radical assay.

## 1. Introduction

The genus *Oenothera* (Onagraceae), also known as Evening Primrose, is native to North and South America [1]. *O. speciosa* Nutt (*syn.* pink primrose, pinkladies and showy evening primrose) is a perennial herb native to the Southeastern United States and Mexico and cultivated in Egypt [2]. Some species are used in traditional medicine to treat various diseases due to their contents of evening primrose oil, which contains a high percentage of γ-linoleic acid [3]. Methanol extracts of different *Oenothera* species have been used as antioxidant [4] and antitumour [5] agents due to their high content of flavonoids and tannins. In the previous literature, tannins [6,7,8,9], phenolic acids [7,10,11,12], flavonoids [7,13,14,15,16] and triterpenes [13] were reported from different *Oenothera* species. The present study aims at isolation and identification of the polyphenols in the aerial parts (leaves and stems) of *O. speciosa* and evaluation of the antihyperglycaemic and anti-inflammatory effects of the total extract, in addition to the antioxidant activity of both the total extract and its major constituents.

## 2. Results and Discussion

**Figure 1:**
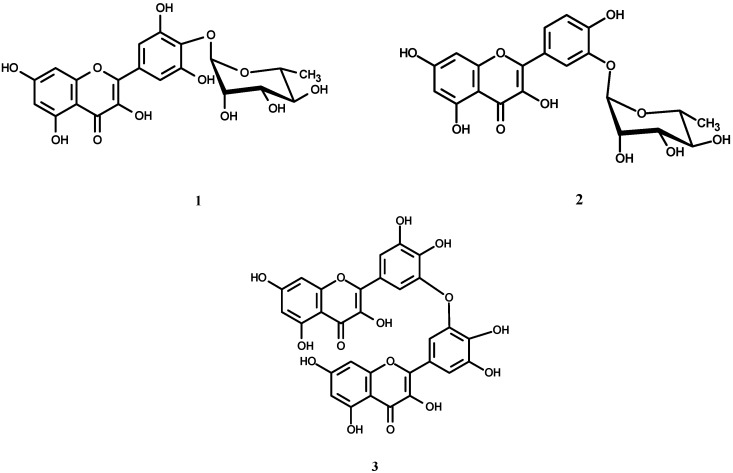
Structures of compounds **1**, **2** and **3**.

The 80 % methanol extract from the aerial parts of *O. speciosa* was fractionated on a polyamide column, followed by consecutive purification steps on cellulose and/or Sephadex LH-20 columns to yield two new flavonol glycosides, **1**, **2 **(Figure 1), and a biflavonol **3 **(Figure 1), together with eleven phenolic metabolites **4-14** [17,18,19,20,21]. In addition, myricetin (**15**), quercetin (**16**) and ellagic acid (**17**) were isolated from the chloroform extract. The structures of all known isolates **4-14** were identified on the basis of interpretation of their chemical, physicochemical analyses (UV, HR-ESIMS, 1D and 2D NMR), comparison with the corresponding published data in the literature [17,18,19,20] and in some cases, comparison with authentic samples for final confirmation.

Based on the chromatographic properties and UV spectral data, **1** and **2** were expected to be myricetin and quercetin *O*-glycosides, respectively [17]. Bathochromic shift in band I (~ + 50 NaOMe) was indicative for substitution of 4'-OH in **1** and 3'-OH in **2** with free 3-OH in both, due to the decomposition in band I [17]. In **1** and **2**, the aglycone moieties were confirmed as by acid hydrolysis that resulted in L-rhamnose in the aqueous phase of both (CoPC). Negative HRESI-MS spectra revealed a molecular ion peak at *m/z* 463.08820 indicating a MF C_21_H_20_O_12_ for **1** and 447.09328 for a MF C_21_H_20_O_11_ for **2**. The ^1^H-NMR of **1** showed a two proton singlet at δ 7.18 for H-2'/6' of 3',4',5'-trihydroxy B-ring, while that of **2** showed an ABX spin coupling system at δ ppm 7.63 (d, *J* = 2.1), 7.53 (dd, *J* = 8.4, 2.1) and 6.87 (d, *J* = 8.4) for H-2', H-6' and H-5' for 3',4'-dihydroxy B-ring and an AM spin coupling system of two meta coupled protons at ~ 6.41 and ~ 6.17, ascribable to H-8 and H-6 of a 5,7-dihydroxy A-ring in both. The location of the rhamnose moiety at 4'-OH in **1** and 3'-OH in **2** was concluded through the intrinsic upfield location of H-1" (δ < 5, [19]) as a broad singlet at 4.9 and CH_3_-6" at 1.08 (d, *J* = 6). These results were further confirmed by ^13^C-NMR, which showed the expected ^13^C-resonances, including the key signals at δ 175.61 (C-4), 145.68 (C-3'/5'), 107.16 (C-2'/6'), for myricetin aglycone in case of **1** [18,21]. The glycosidation of OH-4' was confirmed from the alternative *α*-up/*β*-downfield effects as upfield of C-4' (135.87, Δ -2 ppm), slight downfield of C-3'/5' (145.68, Δ +1.5 ppm), upfield of C-2'/6' (107.16, ~ Δ -2 ppm) and downfield of C-1' at (120.71, Δ +1.5 ppm) [21]. The characteristic upfield location of C-2 at 146.74 (Δ -10 ppm) and downfield location of C-3 at 135.87 (Δ +2.5 ppm) relative to the corresponding 3-*O*-glycosylmyrcetin (**4**, **5**) [18], gave a diagnostic evidence that **1** has a free 3-OH. In case of **2**, the location of rhamnose moiety at 3'-OH was followed from the upfield location of C-3' (145.39) and downfield location of both C-4' (148.22) and C-2' (116.11). As in case of **1**, the presence of a free 3-OH was explained by the intrinsic upfield location of both C-2 (146.23) and C-4 (176.34) and downfield of C-3 (136.22) [18] in comparison with those of myricitrin. Accordingly, **1** was identified as the new myricetin 4'-*O*-α-l-^1^C_4_-rhamnopyranoside, and **2** was established as quercetin 3'-*O*-α-l-^1^C_4_-rhamnopyranoside. 

Compound **3** was expected to be a free flavonol aglycone based on its chromatographic properties (R_f_-values, brilliant yellow fluorescence under UV-light [17]). Negative HRESI-MS showed an oxidized molecular ion peak at *m/z* 615.99829 ascribable to [M-2H]^-^ and corresponding to the MF C_30_H_18_O_15_ and M.wt. of 618.455 amu. This information led us to assign to compound **3** a biflavonol ether structure containing 10 free hydroxyl groups. The corresponding ^1^H-NMR spectrum displayed a splitting pattern of totally aromatic protons of a symmetric biflavonol structure. This was explained as two *meta* coupled doublets, each for two equivalent protons at δ 6.17 and 6.36 with characteristic *J*-value for meta coupling (2.3 Hz). These two meta doublets were assigned to two H-6 and two H-8, respectively of two A-rings having free 5,7-dihydroxy function. In addition, a singlet at 6.91 integrated to two equivalent protons (2 x H-2') of the two B-rings, that upfield shift by the anisotropic effect of diphenyl structure. Also, a singlet integrated to two equivalent protons assigned at δ 7.23 for the two H-6' on both B-rings that hidden by aromatic hydroxyl groups. Depending on all above discussed documents, **3** was expected to be 3',3'-bimyricetin ether. Further confirmation of the structure was achieved from ^13^C-NMR, that showed 15 carbon resonances (each of 2 C) for symmetric biflavonol ether. The presence of free 3-OH groups in both monomeric moieties followed from the characteristic upfield location of C-4 at δ 176.11 and C-2 at 147.62 and downfield location of C-3 at 136.22 [18,21]. These data were completely consistent with the same structural features in ^13^C-NMR spectra of **1** and **2**. The attachment of the two monomers through a 3',3'-di-ether structure was explained due to the upfield location of the signal representing C-3' at 145.75 and downfield of both C-2' and C-4' at 109.06 and 146.10 on both monomer units, respectively. Accordingly, **3** was confirmed as 3',3'-bimyricetin ether (speciin). The structure elucidation was assisted by the comparison with the stimulated ^1^H- and ^13^C-NMR spectra created using the ACD 3.0 NMR program.

The result of LD_50_ determination revealed that the examined extract was non toxic up to a dose of 5 g kg^-1^ b.wt. It was found that the extract exhibited a significant anti-inflammatory activity at a dose of 500 and 1000 mg kg^-1^ 1, 2, 3 and 4 h after carrageenan injection. At a dose of 1000 mg kg^-1^, the extract potency was more or less the same as that of indomethacin (0.92-0.96, Table 1).

**Table 1:** Effect of 80 % aqueous methanol extract of *O. speciosa* aerial parts and indomethacin on carrageenan induced paw oedema in rats (n=6).

Groups	Oedema							
	1 hour		2 hours		3 hours		4 hours	
	% Increase	Potency	% Increase	Potency	% Increase	Potency	% Increase	Potency
Control	96.96 ± 5.6	-----	107.81 ± 8.7	-----	110.03 ± 7.7	-----	110.76 ± 7.9	-----
Methanol extract:	63.96 ± 3.5	0.55	72.48 ± 3.8	0.52	83.15 ± 4.3	0.4	84.49 ± 4.7	0.4
250 mg/kg	(34.03)		(32.77)		(24.43(		(23.72)	
500 mg/kg	45.40 ± 4.2*	0.86	50.76 ± 3.9*	0.84	52.14 ± 5.1*	0.86	52.14 ± 5.1*	0.88
	(53.18)		(52.92)		(52.6)		(51.93)	
1000 mg/kg	41.77 ± 2.5*	0.92	45.78 ± 3.3*	0.92	46.25 ± 4.5*	0.95	46.36 ± 3.8*	0.96
	(56.92)		(57.54)		(57.97)		(58.14)	
Indomethacin	36.96٭± 2.5*	1	40.17± 3.7*	1	42.82± 4.1*	1	43.42± 3.9*	1
	(61.88)		(62.74)		(61.08)		(60.43)	

Data represent the mean value ± S.E. of six rats/group; Data were analyzed using one way ANOVA and Dunnett's multiple comparison test* P<0.05; Percent oedema inhibition (the value in between parenthesis) was calculated as regard to saline control group; Potency was calculated as regard the percentage change of the indomethacin treated group.

**Figure 2:**
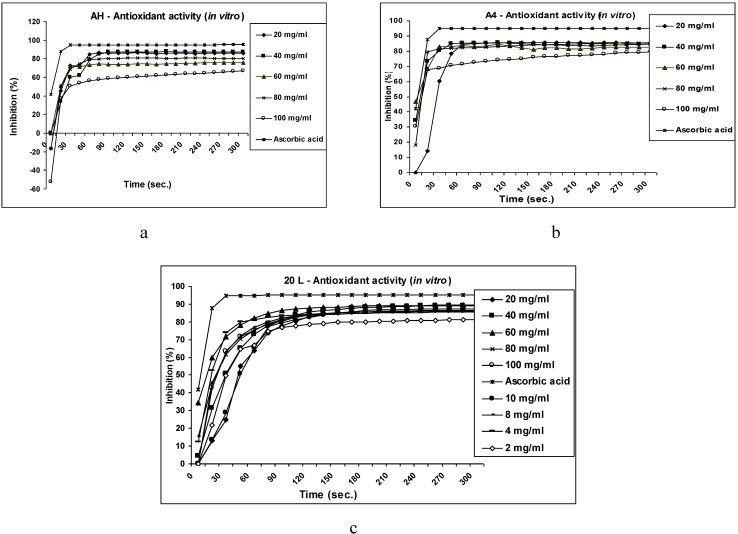
Antioxidant activity *in vitro* using the DPPH radical scavenging activity method. (a) Antioxidant activity of 80 % aqueous methanol extract of *O. speciosa* and ascorbic acid. (b) Antioxidant activity of myricitrin and ascorbic acid. (c) Antioxidant activity of hyperin and ascorbic acid.

The examined extract also exhibited a significant dose dependant antihyperglycaemic effect in streptozotocin induced diabetic rats. This occurs after 2 and 4 weeks treatment at a dose of 250 and 500 mg kg^-1^, as compared with control pre-drug (zero time) for each group and diabetic control in the same time (Table 2). In addition, the examined extract, myricitrin (**4**) and hyperin (**7**) exhibited marked significant scavenging activities against DPPH radicals *in vitro*. The results of the kinetics of the DPPH scavenging reactions of the tested extract and components revealed that the maximum reactive reaction rate after five minutes was 85.9, 87.1, 75.5, 80.5 and 66.6 % for tested extract, 85.1, 84.7, 82.7, 85.8 and 79.7 % for compound **4**, and 81.4, 85.7, 86.2, 86.4, 87.5, 89.2, 89.5, 86.5 and 86.1 % for compound **7**, respectively, at the different concentrations used, in comparison to L-ascorbic acid (95.1 %) (Figure 2a, Figure 2b and Figure 2c).

**Table 2:** Effect of oral administration of 80 % aqueous methanol extract of *O. speciosa* aerial parts on blood glucose levels of diabetic rats induced by streptozotocin injection (n=6).

Group/dosemg/kg b.wt.	Glucose mg/dL in diabetic rats		
	Before treatment	After treatment	
	Zero	15 days	30 days
	X± S.E	X± S.E	X± S.E
Normal control	78.3 ± 3.2	75.4 ± 5.3	72.2 ± 2.8
Diabetic control	390 ± 12.3	385.8 ±11.9 (1.1)	381.4 ±13.3 (2.2)
Methanol extract: 250 mg/kg	419.6 ± 6.7	375.8 ±16.6* (10.4)	347.3 ±13.5*** (17.2)
500 mg/kg	360.5 ± 12.3	300.2 ±13.4**●● (16.7)	250.7±10.4*** ●● (30.5)
Rosiglitazone (0.5 mg/kg)	416.5 ± 14.8	315.6±12.2***● (24.4)	270.5±11.6*** ●● (35.5)

* Statistically significant difference from zero time (* p < 0.05, **p < 0.01, ***p < 0.001). Significantly different from diabetic control: (● p < 0.01, ●● p < 0.001). Percent of change between parentheses was calculated as regard the zero time of each group.

## 3. Experimental

### 3.1. General

The NMR spectra were recorded at 300, 400 and 500 (^1^H) and 75, 100, 125 (^13^C) MHz, on Varian Mercury 300, Bruker APX-400 or JEOL GX-500 NMR spectrometers. The δ-values are reported as ppm relative to TMS in DMSO-*d_6_* (Sigma, St Louis, MO). HRESI-MS analyses were run on LTQ-FT-MS spectrometer (Thermo Electron, 400, Germany). UV analyses for pure samples were recorded, separately, as MeOH (E. Merck, Spectroscopic grade 99.9%) solutions and with different diagnostic UV shift reagents on a JASCO V-630 spectrophotometer (Tokyo, Japan).

### 3.2. Plant material

Aerial parts (leaves and stems) of *O. speciosa* Nutt. were collected in April 2006 from Hamza Botanical Garden, Saft El Laban, Kerdasa Road, Giza, Egypt. Identification of the plant was performed by Dr. Mohamed Gibalie, Lecturer of Taxonomy. A voucher specimen (No: O-1) is kept in the Herbarium of the Pharmacognosy Department, Faculty of Pharmacy, Helwan University, Cairo, Egypt.

### 3.3. Extraction and isolation

The air-dried and powdered leaves and stems (aerial parts) of *O. speciosa* (1.25 kg) were extracted with 80 % hot MeOH (5 x 6 L) under reflux (70 ˚C). After evaporation of the solvent under reduced pressure, the residue (300 g) was extracted with CHCl_3_ under reflux (5 x 1.5 L; 60 ˚C) to yield 5 g dry CHCl_3_ extract and 270 g dry residue. This residue was desalted by precipitation with excess EtOH from water followed by drying of the filtrate in *vacuo* to give 210 g. This residue was suspended in water and chromatographed on a polyamide S (300 g, Fluka, Hannover, Germany) column (Ф 5 x 120 cm) and eluted with H_2_O followed by gradual increase of MeOH portions. Eight collective fractions I-VIII were collected, according to their chromatographic properties (fluorescence-UV light, and responses towards different spray reagents on PC). Fraction I (0-10 % MeOH, 12 g) was phenolic free. Fraction II (20-30 % MeOH, 3.4 g) was fractionated on a Sephadex LH-20 column using H_2_O-MeOH (1:1, v/v) mixture, leading to desorption of two successive subfractions, which were separately collected. Crystallization from hot water of the dry material of the first subfraction yielded pure **13 **(15 mg), while 17 mg of **14** were obtained by crystallization from the 2^nd^ subfraction. Fraction III (30-40 % MeOH, 2.1 g) was purified on a Sephadex column using 40-80 % aqueous MeOH for elution, to give two main subfractions. Each one, containing one major compound, was then purified on a Sephadex column using BIW (butanol-isopropyl alcohol-H_2_O; 4:1:5, upper layer) as eluent to give chromatographically pure samples of **9** (9 mg) and **10** (11 mg). Fraction IV (40-50 % MeOH, 1.89 g) was subjected to repeated CC on Sephadex with BIW, followed by MeOH to give pure samples of **11** (15 mg) and **12** (10 mg). Fraction V (40-50 % MeOH, 3.4 g) was chromatographed on a Sephadex column and eluted with 10-60 % aqueous MeOH to give two main subfractions. The first one eluted with 40 % MeOH contained mainly **7 **and traces of **8**, while the second one contained **8** as the major compound. The first subfraction was then subjected to Sephadex column chromatography using BIW (4:1:5, upper layer) as eluent, resulting in pure **7** (23 mg). Final purification of **8** was achieved through fractionation of the second subfraction on Sephadex column with EtOH as eluent to give pure sample (21 mg). Separation of the individual compounds of fraction VI (50-60 % MeOH, 3.21 g) was carried out on a cellulose column using MeOH-H_2_O (10-60 %) for elution. The first major subfraction was applied on cellulose column and eluted with 40 % aqueous MeOH to give pure sample of **6** (18 mg); and the second one was chromatographed twice on Sephadex column with BIW (4:1:5, upper layer) to give two pure samples of **4** (21 mg) and **5** (25 mg). Fraction VII (60-70 % MeOH, 4.1 g) was subjected to the fractionation on a cellulose column using BIW (4:1:5, upper layer) for elution. Repeated CC of subfractions on Sephadex and MeOH-H_2_O (1:1) and MeOH for elution yielded chromatographic pure samples of **1** (25 mg) and **2** (31 mg). Fraction VIII (80-90 % MeOH, 4.1 g) was chromatographed on a Sephadex column with a step gradient elution of MeOH-H_2_O (20-100 %) yielding 24 mg of **3**. Compounds **15 **(7 mg), **16 **(15 mg) and **17 **(5 mg) were isolated from the chloroform extract by prep-PC on Whatman paper sheets 3 MM (Whatman Ltd., Maidstone, Kent, England) using S_1 _for elution, followed by purification of each desorbed band on a Sephadex column with MeOH as eluent. Sephadex LH-20 (Pharmacia, Uppsala, Sweden), microcrystalline cellulose (E. Merck, Darmstadt, Germany) were used for CC separation, and the fractionation followed up by long UV- light and 2D-PC with solvent systems S_1_: *n*-BuOH-HOAc-H_2_O (4:1:5, upper layer) for first run and S_2_: 15 % aqueous HOAc for second one. All used solvents for separation processes were purchased from El-Nasr Co., Egypt (Analytical grades) except for the organic solvents used for last purification on Sephadex LH-20 columns were purchased from E. Merck (Absolute grades 99.9% MeOH and EtOH).

*Myricetin 4'-O-α-l-rhamnopyranoside* (**1**). Yellow amorphous powder. Yellow fluorescent spot by long UV light, turned to yellow with Naturstoff (Sigma-Aldrich Chemie GmbH, Schnelldorf, Germany) reagent and faint blue with FeCl_3_ (analytical grade, Fluka, Hannover, Germany). R_f_: 0.18 (S_1_), 0.03 (S_2_) on PC; UV/Vis λ_max_ (MeOH): 253, 302, 373; (+NaOMe): 284 sh, 319, 421; (+NaOAc): 325, 385; (+NaOAc/H_3_BO_3_): 339, 390; (+AlCl_3_): 269, 309 sh, 447; (+AlCl_3_/HCl): 269, 314, 449 nm; ^1^H-NMR (300 MHz): 7.18 (2H, s, H-2'/6'), 6.41 (1H, br s, H-8), 6.17 (1H, br s, H-6), 4.81 (1H, br s, H-1"), 1.09 (3H, d, J = 6 Hz, H-6"), 4.10-3.10 (m, remaining sugar protons; ^13^C-NMR (75 MHz): 175.61 (C-4), 164.11 (C-7), 160.52 (C-5), 156.02 (C-9), 146.74 (C-2), 145.68 (C-3'/5'), 135.87 (C-3), 135.69 (C-4'), 120.73 (C-1'), 107.16 (CH-2'/6'), 102.76 (C-10, CH-1"), 98.19 (CH-6), 93.94 (CH-8), 72.34 (CH-4"), 71.47 (CH-2"), 70.35 (CH-3"), 67.61 (CH-5"), 17.94 (CH_3_-6"); Negative HRESI-MS/MS: *m/z* 463.08820 [M-H]^-^, calc. 463.08926 for a MF of C_21_H_20_O_12_, 316.04211 [M-deoxyrhamnoside]^-^.

*Quercetin 3'-O-α-l-rhamnopyranoside* (**2**). Yellow amorphous powder. Yellow fluorescent spot by long UV light turned to yellow with Naturstoff reagent and faint blue with FeCl_3_. R_f_: 0.29 (S_1_), 0.02 (S_2_) on PC; UV/Vis λ_max_ (MeOH): 256, 296 sh, 370; (+NaOMe): 271, 322 sh, 402; (+NaOAc): 271, 318 sh, 389; (+NaOAc/H_3_BO_3_): 264, 389, (+AlCl_3_): 261, 306, 431; (+AlCl_3_/HCl): 262, 306, 430 nm; ^1^H-NMR (300 MHz): 7.63 (1H, d, J = 2.1, H-2'), 7.53 (1H, dd, J = 8.4, 2.1, H-6'), 6.87 (1H, d, J = 8.4, H-5'), 6.42 (1H, d, J = 2.1, H-8), 6.18 (1H, d, J = 2.1, H-6), 4.79 (1H, br s, H-1"), 3.85 (1H, br s, H-2"), 3.7-3.1 (m, remaining sugar protons), 1.09 (3H, d, J = 6.3 Hz, H-6"); ^13^C-NMR (75 MHz): 176.34 (C-4), 164.46 (C-7), 161.18 (C-5), 156.02 (C-9), 148.22 (C-4'), 146.23 (C-2), 145.39 (C-3'), 135.87 (C-4'), 136.21 (C-3), 122.43 (C-1'), 120.51 (CH-6'), 117.04 (CH-5'), 116.11 (CH-2'), 103.47 (C-10), 101.59 (CH-1"), 98.72 (CH-6), 94.53 (CH-8), 72.87 (CH-4"), 72.05 (CH-2"), 70.89 (CH-3"), 68.77 (CH-5"), 18.41 (CH_3_-6"); Negative HRESI-MS/MS: *m/z* 447.09328 [M-H]^-^, calc. 447.09312 for a MF of C_21_H_20_O_11_, 301.03607 [M-deoxyrhamnoside]^-^.

*Speciin* (**3**). Yellow amorphous powder. Shiny yellow fluorescent spot by long UV light intensified by spraying with Naturstoff reagent. R_f_: 0.38 (S_1_), 0.03 (S_2_) on PC; ^1^H-NMR (400 MHz): 7.23 (2H, s, H-6' I, II hidden by aromatic OH groups), 6.91 (2H, s, H-2' I, II), 6.36 (2H, d, J = 2.3 Hz, H-8 I, II), 6.17 (2H, d, J = 2.3, H-6 I, II); ^13^C-NMR (100 MHz): 176.11 (C-4 I, II), 164.25 (C-7 I, II), 161.10 (C-5 I, II), 156.43 (C-9 I, II), 147.62 (C-2 I, II), 146.10 (C-5' I, II, 4' I, II), 145.75 (C-3' I, II), 136.22 (C-3 I, II), 121.13 (C-1', I, II), 109.06 (CH-2' I, II), 107.52 (CH-6' I, II), 103.00 (C-10 I, II), 98.50 (CH-6 I, II), 94.00 (CH-8 I, II); Negative HRESI-MS/MS: *m/z* 615.99829 [M-2H]^-^ for a MF of C_30_H_18_O_15_.

### 3.4. Animals

Adult male pathogen-free Sprague-Dawley rats (120-130 g) and Swiss mice weighing (20-30 g) were purchased from the animal house of National Research Centre and used. The animals were housed in standard metal cages in an air conditioned room at 22 ± 3˚C, 55 ± 5% humidity, and 12 h light and provided with standard laboratory diet and water *ad libitum*. All experimental procedures were conducted in accordance with the guide for care and use of laboratory animals and in accordance with the Local Animal Care and Use Committee. Distilled water was used as a vehicle for extract, while 5 % sodium bicarbonate was used for indomethacin and 0.1 M citrate buffer (pH 4.5) used for streptozotocin (STZ). 

### 3.5. Determination of median lethal dose (LD_50_)

The LD_50_ of the 80 % aqueous methanol extract of *O. speciosa* aerial parts was determined using mice. No percentage mortality was recorded after 24 hours up to a dose of 5 g kg^-1^ and according to Semler [22], who reported that if just one dose level at 5 g kg^-1^ is not lethal, regulatory agencies no longer require the determination of an LD50 value. So the experimental doses used were 1/20, 1/10 and 1/5 of 5 g kg^-1^ of methanol extract of *O. speciosa* aerial parts (250, 500 and 1,000 mg kg^-1^).

### 3.6. Determination of anti-inflammatory activity

Anti-inflammatory activity in acute model was carried out according to the convenient reported method [23]. Rats were divided into five groups each of six, 1^st^ group received orally saline and served as control, 2^nd^, 3^rd^ and 4^th^ groups were given 80 % aqueous methanol extract of *O. speciosa* (250, 500, 1,000 mg kg^-1^), and 5^th^ group was given indomethacin (25 mg kg^-1^, orally) one hour before induction of oedema by subplanter injection of 100 μL of 1% carrageenan (Sigma, USA) in saline into the pad of right paw. The difference in hind footpad thickness was measured immediately before carrageenan injection and 1, 2, 3, 4 h after carrageenan injection with a micrometer caliber [24]. The oedema was expressed as a percentage of change from the control group. 

### 3.7. Determination of antihyperglycaemic activity

The animals were divided into five groups, each of six rats, 1^st^ group received 1 mL saline and served as normal control. Induction of diabetes in the remaining four groups using intra-peritoneal injection of a single dose of 55 mg/kg of streptozotocin (STZ) (MB Biomedical, Inc. France) [25,26], followed by an overnight fast. Diabetes was assessed by determining the blood glucose levels after 72 hrs. The 1^st^ diabetic group received 1 mL saline and served as diabetic control, while the 2^nd^ and 3^rd^ diabetic groups received 80 % aqueous methanol extract (250, 500 mg kg^-1^, orally), the 4^th^ group was treated with the reference antidiabetic drug, rosiglitazone (orally 0.5 mg kg^-1^, GlaxoSmithKline, UK). All groups were treated for four weeks. The blood glucose levels were measured at zero time and after 2 and 4 weeks of treatment by using Biodiagnostic kit of enzymatic colorimetric method, according to Trinder [27].

### 3.8. Determination of antioxidant activity

The 1,1-Diphenyl,2-picryl hy­drazyl (DPPH) radical scavenging activity of *O. speciosa* 80 % aqueous methanol extract (20-100 mg), myricitrin (20-100 mg) and hyperin (2-10, 20-100 mg) was investigated *in vitro*. A methanolic solution of DPPH (2.95 mL) was added to 50 μL samples dissolved in methanol at the different concentrations in a disposable cuvette. The absorbance was measured at 517 nm at regular inter­vals of 15 seconds for 5 minutes. Ascorbic acid was used as a standard (0.1 M) as described by Govindarajan *et al.* [28]. Inhibition was calculated using the following formula:

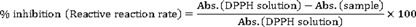



## 4. Conclusions

In summary, a novel biflavonol, named speciin, and two new flavonol glycosides, myricetin 4'-*O*-α-l-rhamnopyranoside and quercetin 3'-*O*-α-l-rhamnopyranoside have been isolated from the 80 % aqueous methanol extract of the aerial parts of *Oenothera speciosa* Nutt. (Onagraceae). In addition, fourteen known phenolic metabolites: myricitrin, europetin 3-*O*-α-l-^1^C_4_-rhamnopyranoside, quercitrin, hyperin, rhamnetin 3-*O*-β-galactopyranoside, caffeic acid, caffeic acid methyl ester, chlorogenic acid, chlorogenic acid methyl ester, gallic acid, gallic acid methyl ester, myricetin, quercetin and ellagic acid have been identified from the plant under investigation. The 80 % aqueous methanol extract exhibited significant antihyperglycaemic and anti-inflammatory activities in dose dependant manner. The investigated extract, myricitrin and hyperin also showed potent *in vitro* antioxidant activity using the DPPH free radical method. 
